# A Comparison of Rice Husks and Peanut Shells as Bedding Materials on Dairy Cows’ Preferences, Behaviour, and Health

**DOI:** 10.3390/ani11071887

**Published:** 2021-06-24

**Authors:** Pengtao Li, Amin Cai, Kris Descovich, Tong Fu, Hongxia Lian, Tengyun Gao, Clive J. C. Phillips

**Affiliations:** 1 College of Animal Science and Technology, Henan Agricultural University, Zhengzhou 450046, China; pengtao_Li@126.com (P.L.); amin_Cai@163.com (A.C.); Futong2004@126.com (T.F.); lhx263@sina.com (H.L.); 2 Center for Animal Welfare and Ethics, School of Veterinary Science, The University of Queensland, Gatton, QLD 4343, Australia; k.descovich1@uq.edu.au; 3 Curtin University Sustainable Policy (CUSP) Institute, Curtin University, Kent St., Bentley, WA 6102, Australia

**Keywords:** dairy cow, preference, behaviour, welfare, peanut shell, rice husk

## Abstract

**Simple Summary:**

Good bedding materials can increase the comfort potential of the lying surface and enhance the welfare of cows in intensive dairy farms. The preference, behaviour, hygiene, and health of cows are affected by different bedding surfaces. In the current study, we evaluated the preference, behaviour, cleanliness, and health of cows on three bedding materials, peanut shells (PS), rice husks (RH), and a combination of two-thirds peanut shells, one-third rice husk (PRC). The daily behaviour, serum metabolites, and productivity of dairy cows were all within normal values, and no statistical differences were found between all three bedding materials, although cows showed a preference for rice husk when given access to all three bedding materials at the same time. Finally, the results suggest that bedding comprised of peanut shells and peanut–rice combinations are all suitable for maintaining the health and comfort of dairy cows.

**Abstract:**

The provision and quality of bedding materials affect the behaviour, welfare, and health of dairy cows. The objective of this study was to evaluate the preference, behaviour, cleanliness, and physiological status of cows on three bedding materials, peanut shells, rice husks, and a combination of two-thirds peanut shells, one-third rice husk. In an initial experiment, 15 nonlactating, pregnant Holstein cows had free access to all 3 bedding treatments for 39 d. Cows spent more time lying down on rice husk (337 min/d) than on peanut–rice combination (212 min/d) and peanut shell (196 min/d) (*p* < 0.05), and lay down most often on rice husk (4.35 bouts/d) than on peanut shell (2.55 bouts/d) (*p* < 0.05) but did not differ between peanut shells and peanut–rice combinations in terms of lying time and lying bouts. In Experiment 2, 12 nonlactating cows were used to assess the effects of the 3 bedding materials on dairy cow behaviour, cleanliness, serum indicators, and productivity. The total duration of lying down (PS: 699.1 min/d, PRC: 645.6 min/d, RH: 852.5 min/d), the frequency of bouts of lying down (PS: 8.7 bouts/d, PRC: 7.6 bouts/d, RH: 11.1 bouts/d), and the mean duration of lying bouts (PS: 83.5 min/bouts, PRC: 91.8 min/bouts, RH: 81.4 min/bouts) did not differ between treatments. Similarly, no differences in eating or drinking behaviour of dairy cows were observed. In terms of hygiene, cleanliness scores did not differ between the three bedding materials, but udder and flank cleanliness decreased and improved, respectively. In addition, treatments did not affect serum metabolites or productivity of the cows. In summary, daily behaviour, serum metabolites, and productivity of dairy cows were all within the normal range, and no statistical differences occurred between the three bedding materials, although cows showed a preference for rice husk when given access to all three bedding materials at the same time. Finally, the results suggest that bedding comprised of peanut shells and peanut–rice combinations are all suitable for maintaining the health and comfort of dairy cows.

## 1. Introduction

Lying down is essential in the daily activities of dairy cows. The duration of time that cows normally spend lying down is between 8 and 16 h each day [1]. Adequate rest is essential for the health and welfare of dairy cows [2]. When cows are deprived of rest, growth hormone and milk yield are reduced [3]. Duration is not the only factor involved in determining the adequacy of rest; the quality of rest is also important and is influenced by bedding characteristics such as the amount, type, and moisture [1,4,5]. Usually, deep, dry, and clean bedding materials are preferred by cows and can improve the welfare of dairy cows [1,5]. The provision of comfortable bedding can improve the health, welfare, and productivity of dairy cows [6,7,8]. The most common types of bedding used in free-stall barns are sand, sawdust, wheat straw, and wood shavings [9]. Nevertheless, it is generally accepted that organic bedding material, such as straw, is favoured by cows over nonorganic materials, such as sand, and that cows lie for longer when offered organic material [4,10,11]. The dry period is a crucial stage in the dairy cow lactation cycle that affects both health and performance after calving [12]. Good facilities for cows during their dry period can promote udder health and increase milk yield in the next lactation [13,14]. Cow lying time after parturition may be linked to health status; for example, ketosis is associated with increased lying time [15]. Consequently, it is very important that dairy cows are carefully monitored and that their environment provides sufficient opportunities for good health and welfare [16].

Rice husk has been used as a bedding material in commercial farms [17], although it has only been tested experimentally for lambs and pigs [18,19,20]. As far as we know, no studies of its suitability for dairy cows have been published, and information on cow preference, health, welfare, and cost would be valuable. In central China, only a small amount of rice is grown, and importation of husks from neighbouring rice-growing regions has a high transportation cost because of its bulk, resulting in a need for alternative bedding materials. Henan grows large quantities of peanuts, with about 1.7 million tons of peanut husks are produced yearly (Henan Academy of Agricultural Sciences Portal; http://www.hnagri.org.cn/, accessed on 5 April 2021). The broken peanut shells are dry and soft, characteristics usually favoured by cows for increased comfort [1,5,21]. Peanut shells also have low amounts of bacteria and moisture, which make them suitable for deep bedding systems [22,23]. Hence, peanut shells provide a potential bedding material for dairy cows in this region.

Scientific studies of dairy cow bedding usually record cow behaviour (e.g., lying, standing, eating and drinking, etc.), body hygiene (particularly of the udder and legs), and health (which may be determined from serum metabolites) [5,15,24]. We explored the characteristics and effects of peanut shells and rice husk in two experiments. In the first, we compared the preference of nonlactating dairy cows for peanut shells, rice husks, or a combination of the two. We examined the physical characteristics of the bedding materials and the influence of ambient weather conditions. In the second experiment, we evaluated the behaviour, cleanliness, serum metabolites, and production of cows provided with the same three bedding treatments.

## 2. Materials and Methods

### 2.1. Experiment 1: The Preference of Dairy Cows for Peanut Shell or Rice Husk Bedding

#### 2.1.1. Animals and Study Design

This study was conducted at the research farm of Henan Agricultural University, in Zhengzhou, China, between November 2019 and January 2020. All procedures involving animals were approved by the animal ethics committee of Henan Agricultural University under the Guidelines for Ethical Review of Laboratory Animal Welfare (GB/T35892). The 55 × 20 m barn had open sides and was naturally lit for 11.5 h each day. The experimental barn orientation was in the east/west direction, and the mean daily temperature at the experiment barn ranged from 0.3 to 18.4 °C. In total, 15 nonlactating and pregnant (mean 37 ± 8 d before parturition (dbp)) Holstein dairy cows between 2 and 6 years old (3 primiparous heifers and 12 multiparous cows) from the research dairies were used. Before the experiment, these cows were kept on a hard bedding surface (dry manure) with other dry cows over 15 days. The barn was divided into three adjacent pens containing the three resting areas by a steel pipe fence, and each resting area (5.9 m long, 5.15 m wide; no cubicle) was freshly filled with a sand base and the following three bedding materials to a depth of 10 cm: (1) peanut shell (PS) (dry matter content (DM): 91.67%); (2) peanut–rice combination (PRC) (a ratio of two parts of peanut shell to one part of rice husk on weight, DM: 91.25% (calculated)); (3) rice husk (RH) (DM: 90.4%). Then, the cows were kept in 3 pens of 5 cows, balanced by parity and calving date, and they had free access to all three bedding types in each pen.

Cows were fed with a total mixed ration at 12 kg/cow (DM basis) containing corn silage, peanut vine, and a commercial concentrate offered daily at 06:00 h. Ad libitum access to water was provided in a trough in each pen. The bedding was levelled daily and faeces were removed while the cows were eating. Fresh bedding material (30 kg) was added to each resting area once weekly. The water trough was emptied, cleaned, and refilled with fresh water daily. Two portable weather stations (detection range of −20–70 °C, 0–100%RH; accuracy ±0.5 °C, ±3% RH) (174H, Testo International Trade (Shanghai) Co., Ltd., Shanghai, China) were installed in the centre of the experimental barn (at a height of 197 cm) to automatically record temperature and relative humidity at 10 min intervals. Data from the portable weather station were downloaded at the end of the test by Testo Comfort Software Basic 5.0 (Testo SE & Co. KGaA, Lenzkirch, Germany).

#### 2.1.2. Preference Test

Four high-definition digital video cameras with built-in infrared lights (MC-8624-H2-400 W, MA CA (China) Co., Ltd., Shenzhen, China) attached to a digital video recorder (MC-8809-K1, MA CA (China) Co., Ltd., Shenzhen, China) were installed in the experimental barn to continuously capture the behaviour of the cows for 43 d. The cows in each pen were tested simultaneously and monitored continuously in individual pens until calving. The first seven days of the experiment were an adaptation period for the cows, and the behaviour was not recorded. In each pen, there were no physical obstacles to prevent the cows from lying down on any of the three bedding surfaces. Behaviour was coded from the video recordings by a trained observer using focal animal sampling with continuous recording, who distinguished individuals by their unique coat colour patches. The following behaviours were recorded: the treatment area in which each cow was lying, the total duration of lying down, the frequency of bouts of lying down, and mean duration of lying bouts (Table 1).

#### 2.1.3. Bedding Material Properties

**Figure 1:**
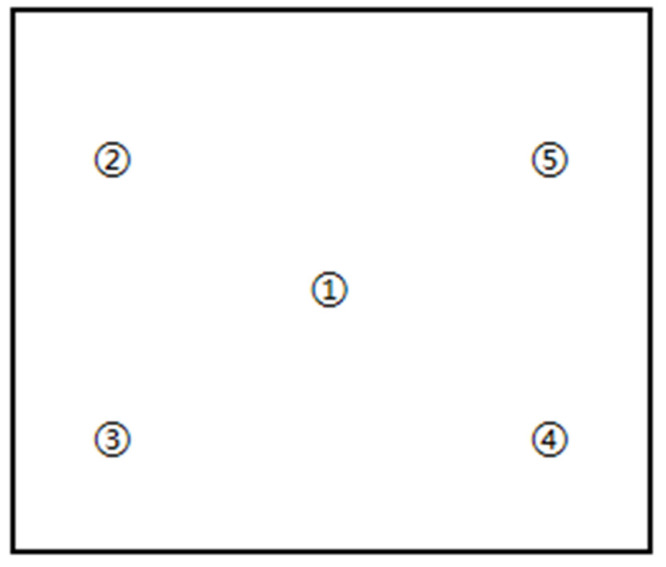
Location of temperature measurement and materials sampling of bedding. 1–5 respectively represent the 5 locations of each bedding surface.

A handheld infrared thermometer (PM6530B, PEAK METER, Shenzhen, China) was used to assess the surface temperature of bedding materials in 5 locations (Figure 1) daily at four times of day (08:00, 10:00, 15:00, and 18:00). To measure the dry matter content of the bedding materials, samples from each bedding area were collected weekly before fresh bedding was added. Each sample consisted of 5 subsamples (about 40 g per sub-sample) from the surface materials (Figure 1). The samples were dried for 72 h at 65 °C using an electric heating constant temperature (blast) drying oven (DHG-9030A, Shanghai Jinghong Experimental Equipment Co., Ltd., Shanghai, China), and the percentage of DM content was calculated as dry weight/wet weight × 100. Infrared thermal images of the bedding materials were collected for three consecutive days by a handheld infrared thermography camera (FLIR C2, FLIR Systems, Inc., Wilsonville, OH, USA) immediately after the cows rose in the morning. Images were then analysed using FLIR researcher software (version 5.13) (FLIR Systems, Inc.) to obtain the mean surface temperature.

### 2.2. Experiment 2: Behaviour, Cleanliness, Health, and Milk Production of Dairy Cows Offered Combinations of Peanut Shells, Rice Husk Bedding, or a Mixture of the Two

#### 2.2.1. Animals and Study Design

In total, 12 nonlactating, pregnant (mean 38 ± 11 dbp) Holstein cows between 2 and 5 years old (7 primiparous heifers and 5 multiparous cows) with no history of mastitis were used. Cows were divided into three bedding treatment groups with the same three bedding materials as used in Experiment 1: peanut shell, peanut–rice combination, and rice husk, balanced for parity (but with two primiparous heifers in the last pen) and calving date. Each treatment group of cows was kept in two pens and was given only one choice of bedding surface to lie down in each pen. The dimensions of the bedding area in each pen were 5.00 m long and 5.15 m wide, with a total area of 25.75 m^2^. Mean daily temperature ranged from −1.2 to 18.1 °C for inside and −1.1 to 19.6 °C for outside of the experiment barn. This research was conducted from January to April 2020 for a total of 60 days.

#### 2.2.2. Cow Behaviour

The same equipment in Experiment 1 was used to monitor the behaviour of dairy cows and environmental conditions. Individual cows in each pen were distinguished by their unique coat colour patches at the time of recording. Behavioural observations and procedures were carried out as per Experiment 1, with an ethogram of behaviour presented in Table 1. The first 7 days of video were not coded to allow the cows to familiarise themselves with the pens and the bedding. Cow behaviour was then monitored until all cows left the pen. Video collected on the day of calving was also not coded because the normal activities of the cow were interrupted.

#### 2.2.3. Cow Cleanliness

**Figure 2:**
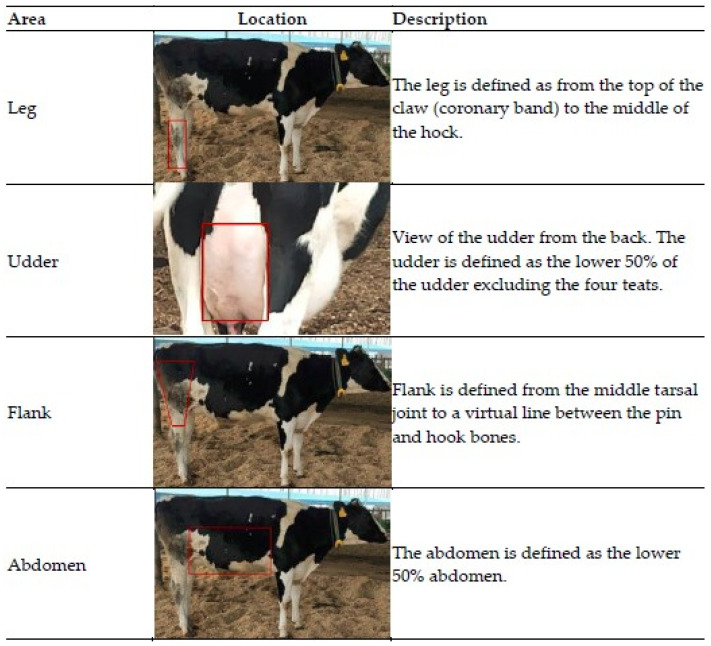
Location of the areas scored using the Dutch Udder Health Centre scheme (UGCN, 2007) and the Canadian Dairy Research Portal for the leg area specifically.

Before cows entered the experimental pen, the cleanliness of their right abdomen, right flank, and udders were separately scored on a scale designed by the Dutch Udder Health Centre (UGCN, 2007) [25]. High ambient relative humidity and muddy pens in winter caused the legs of the cows to be temporarily stained with fresh manure and soil. Therefore, the cleanliness of the cow’s right hind leg was scored before entry into the pen based on a scale from the Canadian Dairy Research Portal (https://www.dairyresearch.ca/cow-comfort.php#self, accessed on 2 January 2021 [26]). The locations of scoring are shown in Figure 2. Hygiene scoring was performed by two trained observers (Table 2). Cows were scored again before leaving the pen, as a factor of period.

#### 2.2.4. Serum Indicators and Productivity

Blood samples were collected from the coccygeal vein into 10 mL tubes without anticoagulants within 24 h of calving and centrifuged at 4000× *g* for 10 min, and the serum collected in a sterile 1.5 mL centrifuge tube and stored at −20 °C until analysis. Serum samples were analysed for serum nonesterified fatty acid (NEFA), serum total calcium (Ca), and B-hydroxybutyrate (BHB) using a fully automated bio-analysis machine (AU5800, Beckman Coulter, Brea, CA, USA). The calves were weighed immediately on a scale (TCS, Sanfeng, Shanghai, China) at birth. The majority of births were assisted by a veterinarian; therefore, dystocia in the cows was not recorded. Cows were milked in an automatic milking system, between 06:00 and 07:00 h, and between 15:00 and 16:00 h. Milk yield for the first seven days after calving was recorded from each cow twice daily using a 28 L glass bottle collecting milk.

#### 2.2.5. Statistical Analysis

Individual cows were considered the observational unit (15 cows for Experiment 1 and 12 cows for Experiment 2). Behaviour results from the single observer were checked for intrarater reliability by that person coding a subset (1 d of all cows) of the same videos twice as a consistency check, using Cohen’s kappa in SPSS (v.22, IBM, Armonk, NY, USA). The average reliability was 0.96. The average interobserver reliability (Cohen’s kappa) for cleanliness scores was 0.773. Assumptions of normality and homoscedasticity were checked using the Shapiro–Wilk test and Levene’s test, respectively, in SPSS. Behaviour, serum metabolites, milk yield, and bedding properties data were analysed using a one-way ANOVA with SPSS. The variables were included as follows: the bedding treatment as an independent variable; all variables as a dependent variable. When the omnibus test was significant, Tukey post hoc tests were conducted to determine which treatments were significantly different from each other. The differences in cleanliness scores within and between treatments over the study were analysed by Kruskal–Wallis and Wilcoxon test. The results are expressed as means and standard errors. Figures were generated with GraphPad Prism 8 software (GraphPad Software, Inc., Sacramento, CA, USA). A statistical difference was assumed if Alpha was <0.05.

## 3. Results

### 3.1. Experiment 1: The Preference of Dairy Cows for Peanut Shell or Rice Husk Bedding

**Figure 3:**
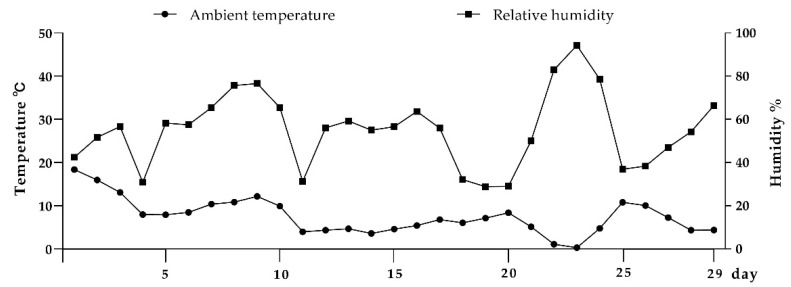
Weather conditions within the barn in Experiment 1.

Due to insufficient device memory, 14 days of weather station data were not recorded. The average daily temperature and relative humidity obtained from the remainder are shown in Figure 3. During the test period, the ambient temperature fluctuated between 0.3 and 18.4 °C with a mean of 7.5 °C. Relative humidity ranged between 28.9 and 94.3% with a mean of 55.1%. The physical properties of the different bedding materials tested in this study are presented in Table 3. The mean DM content for peanut shell, peanut–rice combination, and rice husk were 83.8, 82.5, and 82.7%, respectively. There was no significant difference in DM between bedding types when using sampling times as replicates. No differences were detected between the three bedding treatments for surface temperature. The highest mean temperature from the thermal image of the bedding was observed on the peanut shell (18.0 °C), followed by the peanut–rice combination (15.6 °C) and rice husk (13.5 °C), but there was no significant difference between the three bedding materials (Table 3).

Cows chose to spend more time lying on rice husk (337 min/d) compared with the peanut-rice combination (212 min/d) and peanut shell (196 min/d) and had more frequent lying bouts on rice husk (4.35 bouts/d) than on peanut shell (2.55 bouts/d), but these variables did not differ between peanut shell and peanut–rice combination bedding (Table 4). No differences were observed in the mean duration of lying bouts of cows across the three treatments (Table 4).

### 3.2. Experiment 2: Behaviour, Cleanliness, Health, and Milk Production of Dairy Cows Offered Combinations of Peanut Shell, Rice Husk Bedding, or a Mixture of the Two

Ambient temperature and relative humidity varied throughout the experiment. There were some differences in temperature and humidity between the inside and the outside of the barn. The lowest temperature readings were similar for the two locations (inside: −2.6 °C and outside: −2.8 °C). The maximum temperature was lower inside (25.8 °C) than outside the barn (36.5 °C). Similar minimum (26.4% vs. 24.3%) and maximum (96.4% vs. 96.5%) humidity values were observed in and out the barn.

The total lying time was higher for rice husk than other bedding but no significant differences were found between the three bedding treatments (Table 5). Similarly, the number of lying bouts, mean bout duration, eating time, and drinking time did not differ between the three bedding treatments (Table 5).

Cleanliness scores of cows were not affected by bedding treatment, but the udder cleanliness score increased and the flank cleanliness score descended over the study (Table 6).

Serum metabolite concentrations were all within a normal range, with no statistical differences between bedding materials (Table 7). In addition, serum calcium concentrations of multiparous cows were slightly lower than 4.0 mmol/L, while primiparous cows were higher than 4.0 mmol/L, regardless of bedding treatment (Supplementary Materials Figure S1).

## 4. Discussion

The goal of this study was to compare the preference of dairy cows for three bedding materials and to determine the effects of different bedding on animal behaviour, welfare, and health. During the period when cows had a free choice of three bedding types, cows lied down most often and spent more time lying down on rice husk than on peanut shells and peanut–rice combinations (Table 4), while the average lying time per bout did not differ between the three bedding materials. The lying time, the number of lying bouts, and the mean lying time of cows have been considered indicators of cow preference in previous studies [4,5,26]. The results from the cow behaviour in the current study indicated that rice husk is a preferred bedding material. Cows prefer to spend more time lying down on comfortable, soft, and dry bedding surfaces [21,27]. Wolfe et al. [26] determined that cows spend more time on deep-bedded switchgrass, compared to a combination of switchgrass, water, and lime, because it had the advantage of being softer and drier. Therefore, the properties of bedding materials appear to affect the preference of dairy cows. In the current study, the dry matter content of all three bedding types was higher than 80%, but no differences were detected between bedding materials with a similar dry matter. Organic bedding material with high moisture typically has higher bacterial loads, compared to inorganic material [27,28], and cows prefer to lie down on dry bedding surfaces (DM: 44% vs. 23% and 89.8% vs. 34.7%) [4,21]. In other words, wet bedding damages the welfare of dairy cows by affecting resting time.

On the other hand, the heat distribution of the bedding was elucidated by thermal images when the cows were lying down (Table 3), indirectly reflecting the heat loss of the bedding during resting. In this study, there were no differences in the mean temperature of the thermal images between bedding materials in each pen. For surface temperature, there was no difference between bedding types when compared at the same time. Panivivat et al. [29] found that bedding of wheat straw had higher surface temperatures than rice husk and wood shavings between the months of August and October (summer to autumn in Tennessee, Argentina). However, in the current study, cold ambient temperatures did not cause surface temperature differences to change the preference of cows, which differed from our expectation that the cows would make bedding choices based on the surface temperature of bedding. Therefore, in this research, it appeared that the soft and comfortable nature of rice husk was favoured by the dairy cows.

In Experiment 2, cows were only given one of three potential bedding types. Cow behaviour was unaffected by the type of bedding material presented (Table 5). There were no statistical differences found for any behaviour in response to bedding type. This is in contrast with Experiment 1 in which the cows showed a clear preference for rice husk bedding with longer lying times and more lying bouts, in comparison to peanut shell and peanut–rice combination, when given access to all three types of bedding. This contradiction has also been found in previous studies. Cows have shown a preference for deep-bedded switchgrass and straw bedding over rubber matting when given access to all options, but there was no difference in total lying time when they were only given one of the multiple potential bedding options [26,30]. This suggests that although cows may show a preference for specific bedding materials, it does not always prevent them from resting when faced with only one bedding choice.

Cows tended to spend the most resting time on rice husk and the least time in the peanut–rice combination, but these differences were not significant. This aligns with the number of lying bouts, which was most frequent for the rice husk bedding, followed by peanut shell and the peanut–rice combination (Table 5). In the current study, the total lying time, number of lying bouts and mean duration of lying bouts of cows on the peanut–rice combination and peanut shell fell into the reported normal range of 9.5 to 12.9 h/d, 7 to 10 bouts/d, and 65 to 112 min reported by two previous studies: Ito et al. [31] and Piñeiro et al. [15]. Tucker et al. [2] reported that the lying time of lactating cows is 8 to 13 h/d. Kaufman et al. [32] found that the mean lying time of 10.5 h/d for primiparous cows and 12.3 h/d for multiparous cows at 7 dbp, which is similar to the mean lying time of 11.7 h/d at 10 dbp reported by Huzzey et al. [33]. On the other hand, Solano [34] and Wolfe et al. [26] reported the lying time of lactating cows as 10.4 h/d for straw, 10.6 h/d for sawdust, 10.5 h/d for wood shavings, and 9.9 h/d for switchgrass. In this experiment, the mean lying time was observed to be 10.8 to 14.2 h/d before parturition, regardless of bedding treatments, but no differences were found between the bedding types. However, according to Llonch et al. [24], the daily lying time of dry cows (15.5 h/d) on compost-type bedding was longer than ours. This difference may be attributed to the depth of materials, activity area, and housing system [1,2].

Feed intake is related to animal energy balance [35]. Bedding treatments did not affect the amount of time that cows spent eating or drinking in Experiment 2 of the current study. Beauchemin [36] reported the eating time of lactating cows to be from 2.4 to 8.5 h/d. Hut [37] and Llonch et al. [24] reported the eating time of dry cows to be 5.8 h/d and 2.8 h/d, respectively. Eating times in the current study are largely similar to the published literature, ranging from 4.5 h/d (rice husk bedding) to 5.7 h/d (peanut–rice combination). For drinking time, our results were also in line with the published report (6 min/d) by Llonch et al. [24] in cold weather. Our results indicate that time spent eating and drinking was within the normal range of previous literature. Therefore, the different bedding types assessed in this study still result in normal amounts of eating and drinking behaviour, which is positive for welfare and health.

Cow cleanliness was measured using a Likert scale from 1 (free of dirt) to 4 (very dirty). The cleanliness of cows was not affected by different bedding treatments. Greater improvement in the cleanliness of flanks was found for those cows in post-test than in pre-test, while the udder cleanliness score increased over the study (Table 6). It is well known that as hygiene improves, milk quality also increases and clinical mastitis decreases [38,39,40], and cow cleanliness can be affected by bedding materials and environmental management [4,41]. In the current study, no differences in cleanliness were found between bedding types, which is in line with the reports by Panivivat [29] and Wolfe et al. [26], who compared cows on different bedding materials.

Adequate energetic balance is important for maintaining both cow welfare and adequate production levels. Serum BHB and NEFA concentrations are used as indicators of energy balance and hyperketonemia in cows [42,43]. Ketosis can be diagnosed by a BHB serum concentration of ≥1.2 mmol/L [32,44]. According to Piñeiro et al. [15], the voluntary lying time of cows before calving has a positive quadratic relationship with the NEFA concentration. In the current study, bedding type did not affect the mean BHB and NEFA serum concentrations of the dairy cows (Table 7). Mean BHB and NEFA serum values were within a normal reference range [32,44,45]. Cows suffering from subclinical hypocalcaemia have a higher risk of the displaced abomasum, ketosis, retained placenta, and a higher risk of being culled [46,47]. Furthermore, in severe cases of hypocalcaemia, parturient paresis may develop in dairy cows. According to Reinhardt et al. [48], hypocalcaemia can be defined when total serum calcium concentrations are less than 2.0 mmol/L (8.0 mg/dL) within the first two days after calving. Previous research indicates the prevalence of hypocalcaemia in multiparous cows is higher than for primiparous cows [15,49], which is similar to the finding observed in the current study, that is, the serum calcium concentration of multiparous cows was lower than that of primiparous cows. Milk yield is negatively correlated with lying time [50]. Normocalcemic multiparous cows produce more milk than hypocalcaemia cows, but no association has been found between the lying time of multiparous cows and hypocalcaemia before calving [46,51]. Therefore, it may be expected that milk yield and calf weight could differ between the three bedding treatments, which may be the reason why no significant differences were found between treatments, i.e., because of the lack of difference in lying time. Mean serum concentrations were all within the normal range, suggesting that all three bedding materials are suitable for the welfare and health of dairy cows.

## 5. Conclusions

According to the results of this experimental study, peanut shells and peanut–rice combinations appear to be suitable bedding materials for dairy cows. Daily behaviour, serum metabolites, and productivity measures of the cows were all within the normal range, and no statistical differences were found between bedding types, although cows showed a preference for rice husk when given access to all three bedding materials. Further research is needed to confirm the long-term effect of these deep bedding materials on dairy cow health, welfare, and production.

## Figures and Tables

**Table 1:** Ethogram of cows recorded during Experiments 1 and 2.

Behaviour	Description [24]
Lying down movement	Begins once the cow bends its front carpal joint and lowers the body, and ends when the hindquarter of the cow is completely down and the cow pulls the front leg out from underneath the body.
Lying time	Starts once the ‘Lying down movement’ is complete and ends once the ‘Getting up movement’ commences.
Getting up movement	Begins when the cow lifts the hindquarter from the ground and ends when both front legs touch the ground and the whole-body weight stands on four legs.
Eating	The muzzle or head of the cow is in or over the feed bunk.
Drinking	The muzzle or head of the cow is in or over the water trough.

**Table 2:** Description of cow cleanliness score using scales from the Dutch Udder Health Centre scheme (UGCN) and a scale from the Canadian Dairy Research Portal during Experiment 2.

Score	Description (UGCN)	Description (Canadian)
Score 1	Free of dirt (0% dirt)	Contamination of fresh splashes of manure for <50% of the area
Score 2	Slightly covered with dirt (0 to 10% dirt)	Contamination of fresh splashes of manure for >50% of the area
Score 3	Moderately covered with dirt (10 to 30% dirt)	Contamination of dried caked and fresh manure for >50% of the area
Score 4	Extremely covered with dirt (>30% dirt)	Contamination of entire area with dried caked manure

**Table 3:** Physical properties of peanut shells, a peanut shell–rice husk combination, and rice husks when used as dairy cow bedding treatments.

	Bedding Treatment ^1^					
Item	PS	PRC	RH	SEM	Test Statistic	*p*-Value
^2^ DM, %	83.8	82.5	82.7	0.61	F_(2, 18)_ = 0.42	0.661
^3^ ST at 8:00, °C	−0.04	−0.7	−0.1	0.47	F_(2, 66)_ = 0.17	0.843
ST at 10:00, °C	4.3	4.7	5.4	0.66	F_(2, 66)_ = 0.22	0.806
ST at 15:00, °C	7.2	7.6	8.0	0.52	F_(2, 66)_ = 0.19	0.830
ST at 18:00, °C	3.1	2.7	2.7	0.49	F_(2, 66)_ = 0.06	0.945
^4^ MST, °C	18.0	15.6	13.5	0.91	F_(2, 15)_ = 2.38	0.127

^1^ PS = peanut shell; PRC = peanut-rice combination; RH = rice husk. ^2^ DM = dry matter. ^3^ ST = surface temperature. ^4^ MST = mean surface temperature of thermal image.

**Table 4:** Total lying time, the number of lying bouts, and mean duration of lying bouts for the three bedding treatments during the preference test ^1^.

	Bedding Treatment ^2^					
Item	PS	PRC	RH	SEM	Test Statistic	*p*-Value
Total lying time, min/d	196.4 ^b^	211.9 ^b^	337.3 ^a^	22.73	F_(2, 39)_ = 4.52	0.017
Number of lying bouts, bouts/d	2.55 ^b^	2.78 ^ab^	4.35 ^a^	0.29	F_(2, 39)_ = 4.61	0.016
Mean lying bouts duration, min	53.8	56.6	68.4	3.49	F_(2, 39)_ = 1.71	0.195

^1^ Overall effect was tested by one-way ANOVAs and Tukey tests were used for post hoc pairwise comparisons. ^2^ PS = peanut shells; PRC = peanut–rice combination; RH = rice husk. Different superscript letters within a row indicate *p* < 0.05.

**Table 5:** Effect of three bedding treatments on lying and ingestive behaviour of dairy cows in Experiment 2.

	Bedding Treatment ^1^					
Item	PS	PRC	RH	SEM	Test Statistic	*p*-Value
Total lying time, min/d	699.1	645.6	852.5	43.48	F_(2, 9)_ = 2.640	0.125
Number of lying bouts, bouts/d	8.7	7.6	11.1	0.83	F_(2, 9)_ = 1.837	0.214
Mean lying bouts duration, min	83.5	91.8	81.4	5.69	F_(2, 9)_ = 0.270	0.769
Eating time, min/d	297.7	343.1	267.9	18.40	F_(2, 9)_ = 1.557	0.263
Drinking time, min/d	5.0	3.8	4.2	0.44	F_(2, 9)_ = 0.668	0.536

^1^ PS = peanut shells; PRC = peanut–rice combination; RH = rice husk.

**Table 6:** Effects three bedding materials on the cleanliness scores of cows.

	Bedding Materials ^1^				*p*-Value ^2^	
Item	PS	PRC	RH	SEM	T	*p*
Leg	1.5	1.5	3.0	0.34	0.185	0.470
Udder	1.8	1.5	2.3	0.32	0.506	0.039
Flank	2.3	1.5	2.3	0.25	0.357	0.015
Abdomen	2.3	1.3	1.8	0.30	0.336	0.713

^1^ PS = peanut shells; PRC = peanut–rice combination; RH = rice husk. ^2^ T = treatment effect; *p* = period effect.

**Table 7:** Effects of three bedding materials on serum metabolites, milk yield, and calf weight of cows after calving in Experiment 2.

	Bedding Treatment ^1^					
Item ^2^	PS	PRC	RH	SEM	Test Statistic	*p*-Value
BHB, mmol/L	0.7	0.6	0.9	0.06	F_(2, 8)_ = 2.018	0.195
NEFA, mmol/L	0.7	0.8	0.9	0.09	F_(2, 8)_ = 0.756	0.500
Ca, mmol/L	2.0	2.0	1.9	0.08	F_(2, 8)_ = 0.366	0.704
Milk yield, kg/d	25.7	29.2	28.2	2.26	F_(2, 8)_ = 0.159	0.856
Calf weight, kg	38.0	35.2	35.3	0.94	F_(2, 8)_ = 0.952	0.426

^1^ PS = peanut shells; PRC = peanut–rice combination; RH = rice husk. ^2^ BHB = β-hydroxybutyrate; NEFA = nonesterified fatty acids. Ca = calcium.

## Data Availability

The data used in this study are available from the corresponding author on request.

## Supplementary Materials

The following are available online at https://www.mdpi.com/article/10.3390/ani11071887/s1, Figure S1: Serum calcium concentrations of primiparous and multiparous cows.
