# Supplementary figures and images for: A Comparison of Rice Husks and Peanut Shells as Bedding Materials on Dairy Cows’ Preferences, Behaviour, and Health
Source: Animals (Basel). 2021 Jun 24;11(7):1887. doi: 10.3390/ani11071887 (PMC8300374; doi:10.3390/ani11071887)

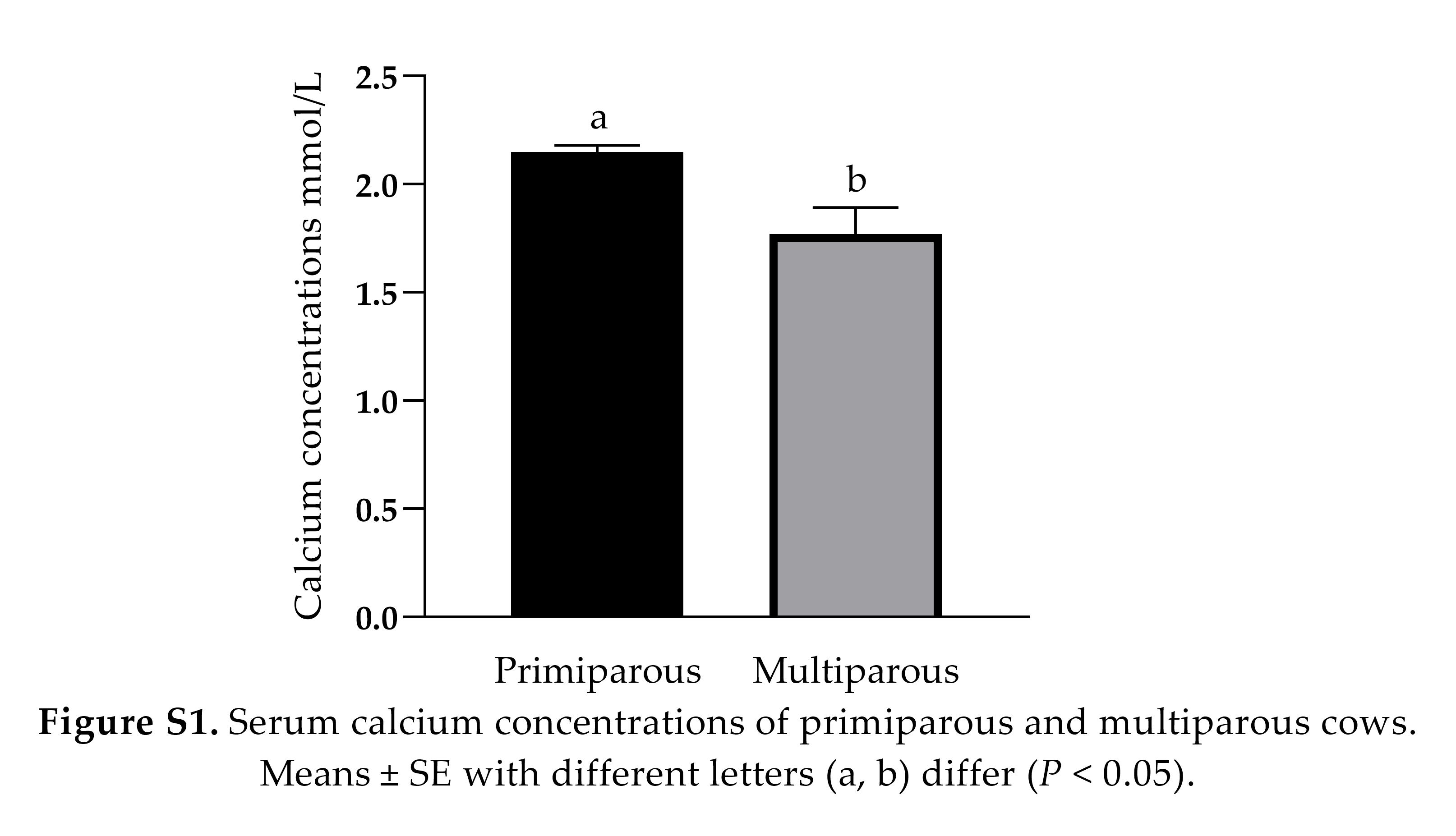

Supplement: Supplementary file 1 [file animals-11-01887-s001.zip › animals-1226729-supplementary.jpg]
